# Triglyceride-glucose index is associated with in-stent restenosis in patients with acute coronary syndrome after percutaneous coronary intervention with drug-eluting stents

**DOI:** 10.1186/s12933-021-01332-4

**Published:** 2021-07-08

**Authors:** Yong Zhu, Kesen Liu, Maolin Chen, Yan Liu, Ang Gao, Chengping Hu, Hong Li, Huagang Zhu, Hongya Han, Jianwei Zhang, Yingxin Zhao

**Affiliations:** 1 Department of Cardiology, Beijing Anzhen Hospital, Capital Medical University, Beijing, 100029 China; 2 Department of Emergency, Beijing Anzhen Hospital, Capital Medical University, Beijing, 100029 China

**Keywords:** Triglyceride-glucose index, Insulin resistance, In-stent restenosis, Drug-eluting stents, Acute coronary syndrome, Percutaneous coronary intervention

## Abstract

**Background:**

The triglyceride-glucose (TyG) index is an alternative marker of insulin resistance (IR) and is closely associated with the prevalence and prognosis of atherosclerotic cardiovascular disease (ASCVD). However, the association between the TyG index and in-stent restenosis (ISR) after drug-eluting stent (DES) implantation in patients with acute coronary syndrome (ACS) remains unknown.

**Methods:**

The present study retrospectively recruited patients who were admitted for ACS and underwent coronary angiography at 6 to 24 months after successful DES-based percutaneous coronary intervention (PCI). In addition, we calculated the TyG index with the following formula: Ln(fasting triglyceride [mg/dL] × fasting blood glucose [mg/dL]/2) and divided patients into 3 groups according to the tertile of the TyG index. Most importantly, multivariate logistic regression analysis models were also constructed to assess the association between the TyG index and DES-ISR in patients with ACS.

**Results:**

A total of 1574 patients with ACS (58.4 ± 9.4 years, 77.4% male) were included in this study. At the median follow-up time of 12 (9–14) months, the prevalence of DES-ISR increased stepwise with the increasing tertile of the TyG index (11.6% vs 17.3% vs 19.4%, p = 0.002), and the TyG index was also higher in the ISR group than in the non-ISR group (9.00 ± 0.58 vs 8.84 ± 0.61, p < 0.001). In addition, the positive association between the TyG index and the prevalence of DES-ISR was also determined in the fully adjusted model (TyG, per 1-unit increase: OR 1.424, 95% CI 1.116 to 1.818, p = 0.005; tertile of TyG, the OR (95% CI) values for tertile 2 and tertile 3 were 1.454 (1.013 to 2.087) and 1.634 (1.125 to 2.374), respectively, with tertile 1 as a reference). The association was also reflected in most subgroups. Moreover, adding the TyG index to the predictive model for DES-ISR in patients with ACS could contribute to an increase in C-statistics (0.675 vs 0.659, p = 0.010), categorical net reclassification improvement (0.090, p < 0.001), and integrated discrimination improvement (0.004, p = 0.040).

**Conclusion:**

An elevated TyG index was independently and positively associated with DES-ISR in patients with ACS who underwent PCI. However, the incremental predictive value of the TyG index for DES-ISR was slight. To further confirm our findings, future studies are needed.

**Supplementary Information:**

The online version contains supplementary material available at 10.1186/s12933-021-01332-4.

## Background

Despite considerable improvement in the anti-restenotic performance of drug-eluting stents (DESs), technological evolution of percutaneous coronary intervention (PCI), medical therapy and other secondary prevention strategies in recent years, in-stent restenosis (ISR) remains a major challenge after PCI, and its occurrence ranges from 3 to 20% [1–3]. The predisposing factors and underlying mechanisms of DES-ISR are complex and remain unclear [3–5]. Therefore, further identifying and controlling the residual risk factors for DES-ISR may have great clinical importance in developing promising strategies for reducing the incidence of DES-ISR.

Insulin resistance (IR), a well-established hallmark of metabolic disorders and systemic inflammation [6], is not only a substantial risk factor for atherosclerotic cardiovascular disease (ASCVD) but also contributes to a worse prognosis [7–9]. Meanwhile, there is also evidence suggesting that IR may play an important role in the development and progression of ISR [10]. However, IR has not received enough attention at present, and the current methods used to assess IR, including hyperinsulinaemia-euglycaemic clamps and homeostatic model assessment (HOMA), are time-consuming, complex, expensive, and not widely available [11, 12]. Therefore, there has been growing interest in determining a reliable, simple, and accessible index to assess IR quantitively.

Accumulating evidence proposes that the triglyceride-glucose (TyG) index, which is derived from fasting triglyceride (TG) and fasting blood glucose (FBG), could serve as a credible and convenient surrogate marker for evaluating IR in clinical practice [12–14]. Mounting evidence has demonstrated that the TyG index is significantly associated with an increased risk of diabetes mellitus (DM), hypertension, metabolic syndrome, atherosclerosis, and even the progression of atherosclerosis [15–19]. Furthermore, recent data also confirmed that the TyG index is strongly associated with worse long-term prognosis and could be used to optimize early risk stratification in patients with coronary artery disease (CAD) [20, 21].

However, to the best of our knowledge, the association between the TyG index and DES-ISR remains unknown, and no relevant studies have been designed to investigate this topic to date. To address this knowledge gap, we performed the present study to investigate the association between the TyG index and ISR in patients with acute coronary syndrome (ACS) after successful DES-based PCI.

## Methods

### Study population

Patients who were diagnosed with ACS and underwent follow-up angiography ranging from 6 to 24 months after successful PCI from January 2018 to August 2020 in Beijing Anzhen Hospital, Capital Medical University, were reviewed retrospectively. The main exclusion criteria were as follows: (1) age less than 18 years; (2) history of coronary artery bypass grafting (CABG); (3) culprit lesion treated with bare metal stent (BMS)/only underwent balloon angioplasty without DES implantation; (4) suspected familial hypertriglyceridaemia (triglyceride ≥ 5.65 mmol/L); (5) severe hepatic and renal dysfunction (estimated glomerular filtration rate (eGFR < 30 mL/min/1.73 m^2^)); and (6) acute/chronic inflammatory disease, malignancy, and body mass index (BMI) ≥ 45 kg/m^2^. Most importantly, this retrospective study was performed in line with the Declaration of Helsinki and was approved by the Ethics Committee of Beijing Anzhen Hospital, Capital Medical University. Additionally, written/oral informed consent was also obtained from the participants.

### Intervention and management

Coronary intervention and periprocedural management were performed according to current guidelines in our centre [22]. Before intervention, all enrolled patients were prescribed a loading dose of aspirin (300 mg) and clopidogrel (300 mg) or ticagrelor (180 mg). In addition to antiplatelet therapy, anticoagulation with unfractionated heparin (70–100 IU/kg, with an additional bolus if necessary) was also used to maintain an activated clotting time > 250 s. During the intervention, radial access was regarded as the standard approach, unless there were overriding procedural considerations. The application of intracoronary imaging, type of second-generation DES, and size of stents were all at the discretion of the operator. Following the intervention, secondary prevention strategies recommended by current guidelines were also prescribed to patients.

### Data collection and definitions

Patient demographics and clinical characteristics, including age, sex, smoking status, previous medical history, left ventricular ejection fraction (LVEF), angiographic evaluation results, procedure details, and medication used at discharge, were all collected from the electronic medical recording system by trained physicians who were blinded to the aim of the study. In addition, peripheral venous blood samples were obtained after overnight fasting (> 8 h) for analysis. Then, FBG, uric acid, eGFR, high sensitivity-C reactive protein (Hs-CRP) and serum lipid profiles, including TG, total cholesterol (TC), low-density lipoprotein-C (LDL-C), and high-density lipoprotein-C (HDL-C), which were determined in the central laboratory of Beijing Anzhen Hospital, Capital Medical University, were also recorded.

The TyG index was determined with the following formula: Ln (fasting TG [mg/dL] × FBG [mg/dL]/2) [14], and BMI was calculated as weight (kg)/height squared (m^2^). Smoking status was stratified into 3 levels: never, former (quit smoking > 1 month), and current. Hypercholesteraemia was defined as a fasting serum TC > 6.22 mmol/L, LDL-C > 4.14 mmol/L or treatment with lipid-lowering drugs [23]. The diagnosis of DM was confirmed by a previous history of DM, active treatment with antidiabetic medication, or the typical symptoms of DM with FBG > 7 mmol/L and/or random blood glucose > 11.1 mmol/L [24]. Additionally, based on angiographic evaluation results, multivessel disease was defined as ≥ 2 vessels with significant diameter stenosis (≥ 50%). Chronic total occlusion (CTO) was defined as completed obstruction of a native coronary artery ≥ 3 months.

### Follow-up angiography and evaluation of ISR

All the patients included in the analysis underwent follow-up angiography using the standard Judkin technique at 6 to 24 months after successful PCI. According to angiographic follow-up results, patients were divided into the non-ISR group or ISR group, which was defined as the presence of significant diameter stenosis (≥ 50%) at the segment inside the stent or involving its 5-mm edges, which is in line with previous studies [25, 26]. Of note, the follow-up angiography was interpreted by 2 independent and experienced cardiologists who were unaware of the patients’ information. Discrepancies encountered in the processes of identifying ISR were resolved by discussion with a senior researcher.

### Statistical analysis

Participants recruited were mainly classified based on the tertile of the TyG index in the present study. To summarize the clinical characteristics of participants, continuous variables were expressed as the mean ± standard deviation or the median with interquartile range depending on the normality of the data distribution, and categorical variables were presented as absolute values (percentages). The differences in continuous variables with a normal distribution across the TyG tertile were compared with one-way analysis of variance. For continuous parameters with skewed distribution, the Kruskal–Wallis H test was performed to detect differences. Differences in categorical variables were analysed by the chi-square test or Fisher’s exact test. In addition, the association between the TyG index and other cardiometabolic risk factors was also assessed by using Pearson’s correlation test or Spearman’s rank test when appropriate.

To identify determinants of ISR in patients after successful PCI with DES, univariate logistic regression analysis was performed. The baseline variables were selected and included in the multivariable logistic regression analysis model if they showed p < 0.1 in univariate analysis or were clinically relevant to DES-ISR. Finally, 3 models were established to control confounding variables and evaluate the association between the TyG index (modulated as continuous or categorical variables) and DES-ISR. Model 1 was adjusted for age, sex, and BMI; model 2 was adjusted for variables included in model 1 plus LVEF, Hs-CRP, hypertension, DM, and previous PCI; and model 3, which is the fully adjusted model, was adjusted for variables in model 2 plus SYNTAX score, target vessel in the left anterior descending artery (LAD), target vessel in the right coronary artery (RCA), intracoronary imaging, DES-sirolimus, total length of stents, and minimal stent diameter.

Furthermore, to evaluate the predictive value of the TyG index for DES-ISR, the area under the curve (AUC) and the optimal cut-off value were assessed through receiver operating characteristic (ROC) curve analysis. Meanwhile, to evaluate whether introducing the TyG index into the model of established risk factors could improve the predictive value, the C-statistic was calculated and compared by De-Long’s test. Additionally, the categorical net reclassification improvement (NRI) and integrated discrimination improvement (IDI) were also calculated to further evaluate the incremental predictive value of the TyG index.

All statistical analyses in the present study were performed with SPSS 20.0 (IBM, Armonk, New York), R Programming Language 4.0.2, and MedCalc 19.1 (MedCalc software, Belgium). Most importantly, statistical significance was regarded as a two-sided p value < 0.05.

## Results

### Baseline characteristics

A total of 1574 patients who underwent follow-up angiography at a median follow-up time of 12 (9–14) months after successful DES-based PCI were enrolled. As shown in Table 1, the mean age of the study population was 58.40 ± 9.40 years old, and 1218 (77.4%) participants were male. The prevalence of current smoking, hypertension, DM, and previous PCI were 35.3%, 64.5%, 34.6% and 18.4%, respectively. Regarding angiographic findings, multivessel/left main artery (LM) disease (75.9%) was common, and half of the patients had implanted multiple stents (≥ 2).

**Table 1:** Baseline characteristics of patients stratified by tertile of TyG index

	Total (n = 1574)	Tertile of TyG index			P-value
		I (Lowest) (n = 525)	II (Median) (n = 533)	III (Highest) (n = 516)	
		6.55 < TyG ≦ 8.58	8.58 < TyG ≦ 9.11	9.11 < TyG ≦ 10.89	
Age, years	58.40 ± 9.40	59.54 ± 9.23	58.94 ± 9.46	56.67 ± 9.28	< 0.001
Male, n (%)	1218 (77.4)	423 (80.6)	406 (76.2)	389 (75.4)	0.097
BMI, kg/m^2^	25.96 ± 3.23	25.28 ± 3.06	26.04 ± 3.18	26.57 ± 3.32	< 0.001
LVEF, %	62.17 ± 7.01	62.55 ± 6.61	61.93 ± 7.14	62.04 ± 7.26	0.312
Diagnosis, n (%)					0.061
UA	1334 (84.8)	459 (87.4)	452 (84.8)	423(82.0)	
NSTEMI	116 (7.4)	33 (6.3)	44 (8.3)	39 (7.6)	
STEMI	124 (7.9)	33 (6.3)	37 (6.9)	54 (10.5)	
Medical history, n (%)					
Current smoking	556 (35.3)	173 (33.0)	184 (34.5)	199 (38.6)	0.030
Hypertension	1016 (64.5)	313 (59.6)	344 (64.5)	359 (69.6)	0.004
Hypercholesteraemia	610 (38.8)	192 (36.6)	198 (37.1)	220 (42.6)	0.086
Diabetes mellitus	544 (34.6)	111 (21.1)	173 (32.5)	260 (50.4)	< 0.001
Previous stroke	127 (8.1)	46 (8.8)	43 (8.1)	38 (7.4)	0.710
Previous PCI	289 (18.4)	95 (18.1)	108 (20.3)	86 (16.7)	0.317
Laboratory tests					
Hs-CRP, mg/L	1.43 (0.56, 3.97)	0.93 (0.40, 3.11)	1.44 (0.59, 3.78)	1.89 (0.82, 4.58)	< 0.001
eGFR, mL/min/1.73 m^2^	96.54 ± 14.72	96.71 ± 13.93	96.56 ± 14.46	96.34 ± 15.78	0.921
Uric acid, umol/L	345.49 ± 84.69	332.35 ± 74.62	345.42 ± 81.91	358.92 ± 94.61	< 0.001
Homocysteine, umol/L	12.50 (9.70, 16.10)	12.80 (9.80, 16.55)	12.70 (9.85, 16.1)	12.00 (9.50, 15.68)	0.095
FBG, mmol/L	6.57 ± 2.31	5.50 ± 0.97	6.19 ± 1.54	8.04 ± 3.06	< 0.001
Triglycerides, mmol/L	1.40 (1.02, 1.98)	0.90 ± 0.26	1.47 ± 0.33	2.47 ± 0.88	< 0.001
TC, mmol/L	4.11 ± 1.07	3.75 ± 0.92	4.09 ± 0.97	4.50 ± 1.17	< 0.001
HDL-C, mmol/L	1.07 ± 0.24	1.15 ± 0.26	1.06 ± 0.23	0.99 ± 0.21	< 0.001
LDL-C, mmol/L	2.47 ± 0.89	2.24 ± 0.82	2.51 ± 0.86	2.67 ± 0.93	< 0.001
Angiography					
LM disease, n (%)	139 (8.8)	53 (10.1)	50 (9.4)	36 (7.0)	0.179
Multivessel/LM disease, n (%)	1195 (75.9)	384 (73.1)	423 (79.4)	388 (75.2)	0.055
Chronic total occlusion, n (%)	367 (23.3)	104 (19.8)	130 (24.4)	133 (25.8)	0.058
SYNTAX score	13.93 ± 7.42	13.72 ± 7.83	14.36 ± 7.43	13.70 ± 6.97	0.253
Intervention					
Target vessel, n (%)					
LM	78 (5.0)	30 (5.7)	30 (5.6)	18 (3.5)	0.175
LAD	889 (56.5)	304 (57.9)	296 (55.5)	289 (56.0)	0.714
LCX	434 (27.6)	125 (23.8)	151 (28.3)	158 (30.7)	0.042
RCA	640 (40.7)	214 (40.8)	217 (40.7)	209 (40.5)	0.996
Intracoronary imagine, n (%)	100 (6.4)	40 (7.6)	31 (5.8)	29 (5.6)	0.343
DES-sirolimus, n (%)	849 (53.9)	288 (54.9)	281 (52.7)	280 (54.3)	0.772
DES-zotarolimus, n (%)	345 (21.9)	127 (24.2)	117 (22.0)	101 (19.6)	0.198
DES-everolimus, n (%)	597 (37.9)	187 (35.6)	210 (39.4)	200 (38.8)	0.400
Number of stent, /patients	2.00 (1.00, 2.00)	1.00 (1.00, 2.00)	2.00 (1.00, 2.00)	1.00 (1.00, 2.00)	0.227
Multiple stents (n ≥ 2)	789 (50.1)	256 (48.8)	280 (52.5)	253 (49.0)	0.392
Length of stents, mm/patients	36 (23, 61)	36 (21, 58.50)	38 (23, 62)	35 (23, 63.75)	0.223
Minimal stent diameter, mm	2.84 ± 0.46	2.88 ± 0.47	2.83 ± 0.46	2.82 ± 0.46	0.105
Medications at discharge, n (%)					
Aspirin	1574 (100.0)	525 (100.0)	533 (100.0)	516 (100.0)	> 0.99
Clopidogrel/Ticagrelor	1574 (100.0)	525 (100.0)	533 (100.0)	516 (100.0)	> 0.99
Statin	1570 (99.7)	523 (99.6)	532 (99.8)	515 (99.8)	0.779
β-block	1088 (69.2)	337(64.2)	378 (71.1)	373 (72.3)	0.009
ACEI/ARB	722 (45.9)	206 (39.2)	250 (46.9)	266 (51.6)	< 0.001
Insulin	174 (11.1)	37 (7.0)	52 (9.8)	85 (16.5)	< 0.001
Other hypoglycemic agents	431 (27.4)	86 (16.4)	133 (25.0)	212 (41.1)	< 0.001

TyG: triglyceride-glucose index; BMI: body mass index; LVEF: left ventricular ejection fraction; UA: unstable angina; NSTEMI: non ST-segment elevation myocardial infarction; STEMI: ST-segment elevation myocardial infarction; PCI: percutaneous coronary intervention; Hs-CRP: high sensitivity-C reactive protein; eGFR: estimated glomerular filtration rate; FBG: fasting blood glucose; TC: total cholesterol; HDL-C: high-density lipoprotein-C; LDL-C: low-density lipoprotein-C; LM: left main artery; LAD: left anterior descending artery; LCX: left circumflex artery; RCA: right coronary artery; DES: drug-eluting stent; ACEI/ARB: angiotensin-converting enzyme inhibitor/angiotensin receptor blocker

Based on the tertile of the TyG index, the patients recruited were further divided into 3 groups (Table 1). As demonstrated in Table 1, serum Hs-CRP, uric acid, FBG, TG, TC, and LDL-C; the prevalence of current smoking, hypertension, and DM; and BMI were all significantly increased with increasing TyG index tertile. Meanwhile, the patients in the higher TyG index group also had a trend for a higher proportion of multivessel/LM disease and CTO. Nevertheless, the patients with a higher TyG index were relatively younger and had a lower HDL-C.

Additionally, the differences between the ISR (n = 253) and non-ISR groups were also analysed (Additional file 1: Table S1). As revealed in Additional file 1: Table S1, patients in the ISR group were more likely to have DM and previous PCI, have higher concentrations of Hs-CRP and FBG, and suffer from more severe CAD. For the procedure details, those with ISR were more likely to undergo PCI for LAD and RCA lesions, use DES-sirolimus, and have longer total length of stents. However, PCI under the guidance of intracoronary imaging was more common in those without ISR.

### Association between the TyG index and other cardiometabolic risk factors

To further identify the association between the TyG index and other cardiometabolic risk factors, Spearman’s rank or Pearson’s correlation analysis was performed, and the results are described in Table 2. The TyG index correlated positively with BMI, Hs-CRP, uric acid, TC, and LDL-C. In contrast, the TyG index was negatively associated with age (r = − 0.154, p < 0.001) and HDL-C (r = − 0.300, p < 0.001).

**Table 2:** Association between TyG index and other cardiometabolic risk factors

Variables	Correlation coefficient (r)	P-value
Age	− 0.154	< 0.001
BMI	0.174	< 0.001
Hs-CRP	0.167	< 0.001
eGFR	− 0.012	0.648
Uric acid	0.123	< 0.001
TC	0.293	< 0.001
HDL-C	− 0.300	< 0.001
LDL-C	0.194	< 0.001
Homocysteine	− 0.044	0.083

TyG: triglyceride-glucose index; BMI: body mass index; Hs-CRP: high sensitivity-C reactive protein; eGFR: estimated glomerular filtration rate; FBG: fasting blood glucose; TC: total cholesterol; HDL-C: high-density lipoprotein-C; LDL-C: low-density lipoprotein-C

### TyG index and the prevalence of ISR after successful DES-based PCI

**Fig. 1:**
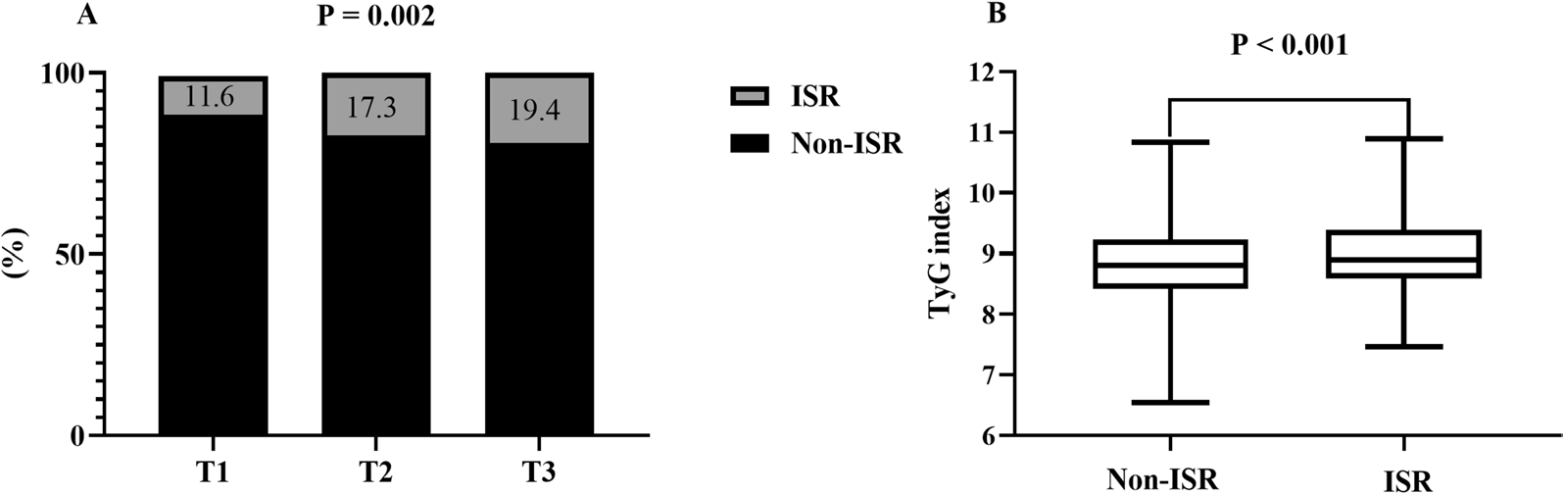
The impacts of the TyG index on the prevalence of DES-ISR (**A**) and comparison of the TyG index level between the ISR and non-ISR groups (**B**) in the overall study population. TyG index: triglyceride-glucose index; DES: drug-eluting stent; ISR: in-stent restenosis

As shown in Fig. 1A, the prevalence of ISR had stepwise increase with the increasing tertile of the TyG index (11.6% vs 17.3% vs 19.4%, p = 0.002). Additionally, it is noteworthy that the ISR group also had a significantly higher TyG index than the non-ISR group (9.00 ± 0.58 vs 8.84 ± 0.61, P < 0.001, Fig. 1B).

**Fig. 2:**
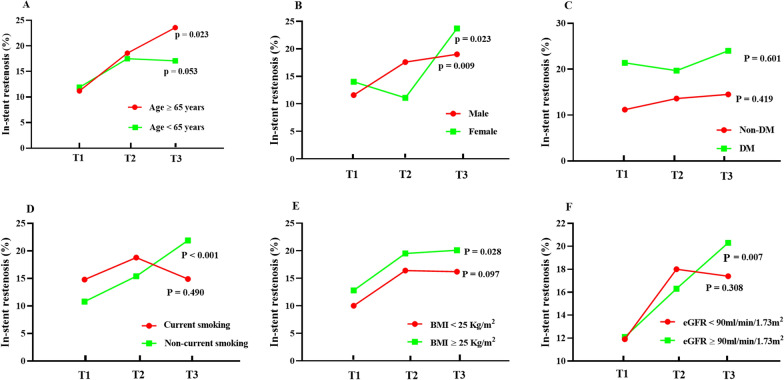
The impact of the TyG index on the prevalence of DES-ISR across subgroups of age (**A**), sex (**B**), DM status (**C**), current smoking status (**D**), dichotomized baseline BMI (**E**), and dichotomized baseline eGFR (**F**). TyG index: triglyceride-glucose index; DES: drug-eluting stent; ISR: in-stent restenosis; DM: diabetes mellitus; BMI: body mass index; eGFR: estimated glomerular filtration rate

The impacts of the TyG index on the prevalence of ISR were also analysed across the different subgroups. As demonstrated in Fig. 2, the subgroup analysis demonstrated that the prevalence of ISR was increased across the tertiles of the TyG index in both male (11.6% vs 17.6% vs 19.0%, p = 0.009) and female (14.0% vs 11.1% vs 23.7%, p = 0.023) subjects. In addition, a trend towards a higher percentage of ISR across the tertiles of the TyG index was also observed in the subgroups of age ≥ 65 years (11.2% vs 18.6% vs 23.6%, p = 0.023), noncurrent smoking (10.8% vs 15.4% vs 21.9%, p < 0.001), BMI ≥ 25 kg/m^2^ (12.8% vs 19.5% vs 20.1%, p = 0.028), and eGFR ≥ 90 mL/min/1.73 m^2^ (12.1% vs 16.3% vs 20.3%, p = 0.007). Meanwhile, comparisons of the TyG index between patients with or without ISR in those subgroups were also analysed and are presented in Additional file 1: Figure S1.

### Association of the TyG index and the risk of DES-ISR in univariate analysis and multivariable analysis

In univariate logistic regression analysis, the TyG index (per 1-unit increase), as a continuous variable, had a positive association with the risk of ISR after successful DES-based PCI (OR = 1.522, 95% CI 1.222 to 1.895, p < 0.001). When the TyG index was modulated as a categorical variable and the tertile 1 group was used as a reference, the incidence of DES-ISR was elevated in the tertile 2 (OR = 1.587, 95% CI 1.119 to 2.249, p = 0.009) and tertile 3 groups (OR = 1.828, 95% CI 1.295 to 2.581). Meanwhile, DES-ISR also correlated significantly with some other variables in the univariate analysis, which is demonstrated in Additional file 1: Table S2.

**Fig. 3:**
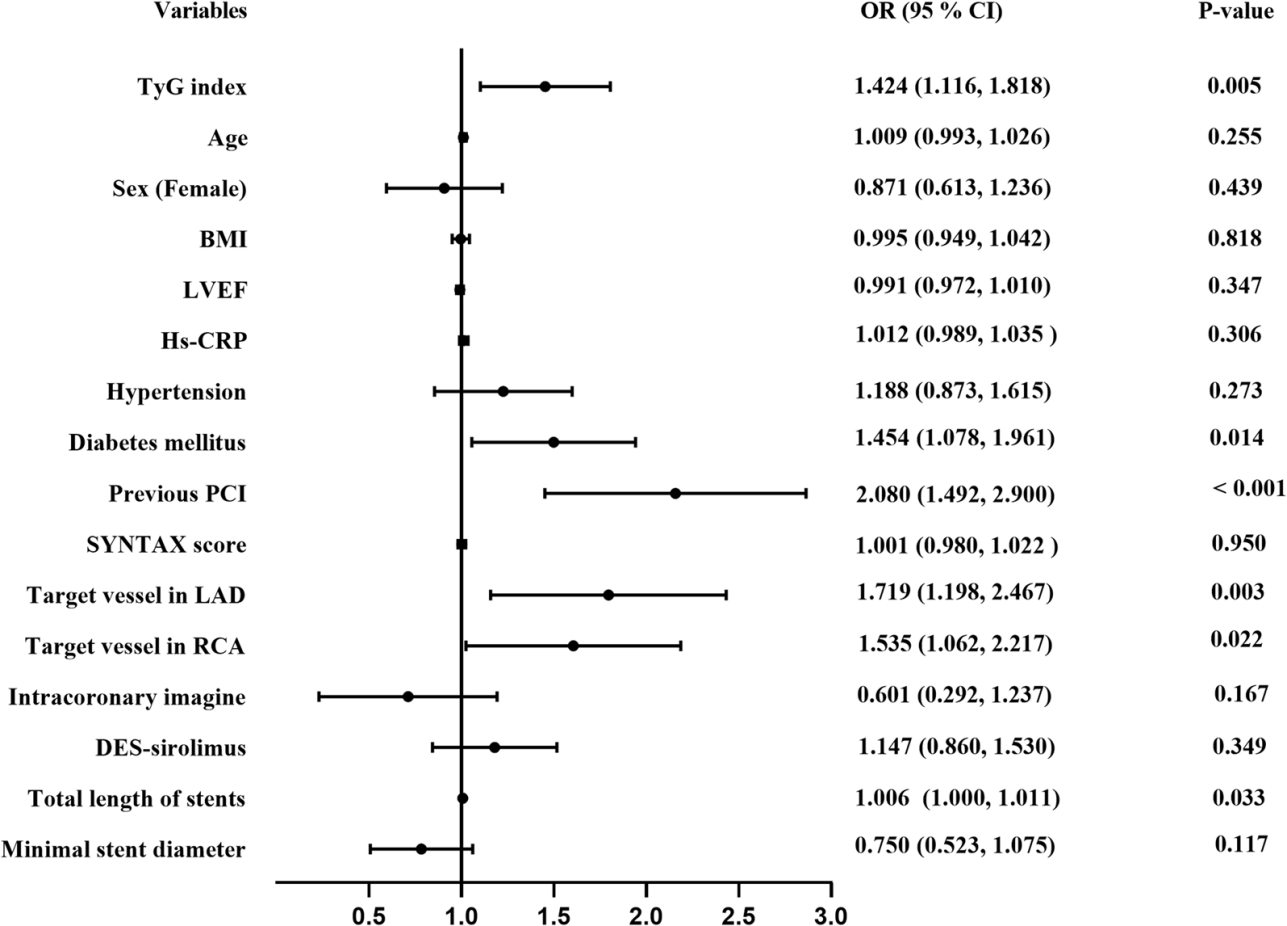
Forest plot of the multivariable logistic regression analysis model in patients with ACS exploring the association between the TyG index and DES-ISR. ACS: acute coronary syndrome; TyG index: triglyceride-glucose index; DES: drug-eluting stent; ISR: in-stent restenosis; BMI: body mass index; LVEF: left ventricular ejection fraction; Hs-CRP: high sensitivity-C reactive protein; PCI: percutaneous coronary intervention; LAD: left anterior descending artery; RCA: right coronary artery; OR: odds ratio; CI: confidence interval

In multivariable logistic regression models, the TyG index was modulated as a continuous variable first. As presented in Table 3 and Fig. 3, a 1-unit increase in the TyG index was independently associated with an increased risk of DES-ISR in model 1 (OR = 1.569, 95% CI 1.253 to 1.965, p < 0.001), model 2 (OR = 1.396, 95% 1.101 to 1.711, p = 0.006), and even in the fully adjusted model (model 3) (OR = 1.424, 95% CI 1.116 to 1.818, p = 0.005). When the TyG index was evaluated as a tertile, the association persisted in the 3 models (Table 3). As shown in Table 3, after fully adjusting for the potential confounding factors in model 3, the adjusted OR (95% CI) values for patients in tertile 2 and tertile 3 were 1.454 (1.013 to 2.087, p = 0.043) and 1.634 (1.125 to 2.374, p = 0.010), respectively, compared to reference tertile 1.

**Table 3:** Association of TyG index with DES-ISR in multivariable logistic regression models

	OR	95% CI	P-value
Model 1			
TyG, per 1-unit increase	1.569	1.253 to 1.965	< 0.001
Tertile 1	Reference		
Tertile 2	1.599	1.125 to 2.272	0.009
Tertile 3	1.890	1.328 to 2.688	< 0.001
Model 2			
TyG, per 1-unit increase	1.396	1.101 to 1.711	0.006
Tertile 1	Reference		
Tertile 2	1.473	1.031 to 2.104	0.033
Tertile 3	1.619	1.120 to 2.339	0.010
Model 3			
TyG, per 1-unit increase	1.424	1.116 to 1.818	0.005
Tertile 1	Reference		
Tertile 2	1.454	1.013 to 2.087	0.043
Tertile 3	1.634	1.125 to 2.374	0.010

Model 1: adjusted for age, sex, and BMI

Model 2: adjust for age, sex, BMI, LVEF, Hs-CRP: hypertension, diabetes mellitus, and previous PCI

Model 3: adjust for age, sex, BMI, LVEF, Hs-CRP, hypertension, diabetes mellitus, previous PCI, SYNTAX score, target vessel in LAD, target vessel in RCA, the application of intracoronary imagine; DES-sirolimus; total length of stents, and minimal stent diameter

TyG: triglyceride-glucose index; DES: drug-eluting stents; ISR: in-stent restenosis; OR: odds ratio; CI: confidence interval; BMI: body mass index; LVEF: left ventricular ejection fraction; Hs-CRP: high sensitivity-C reactive protein; PCI: percutaneous coronary intervention; LAD: left anterior descending artery; RCA: right coronary artery

**Fig. 4:**
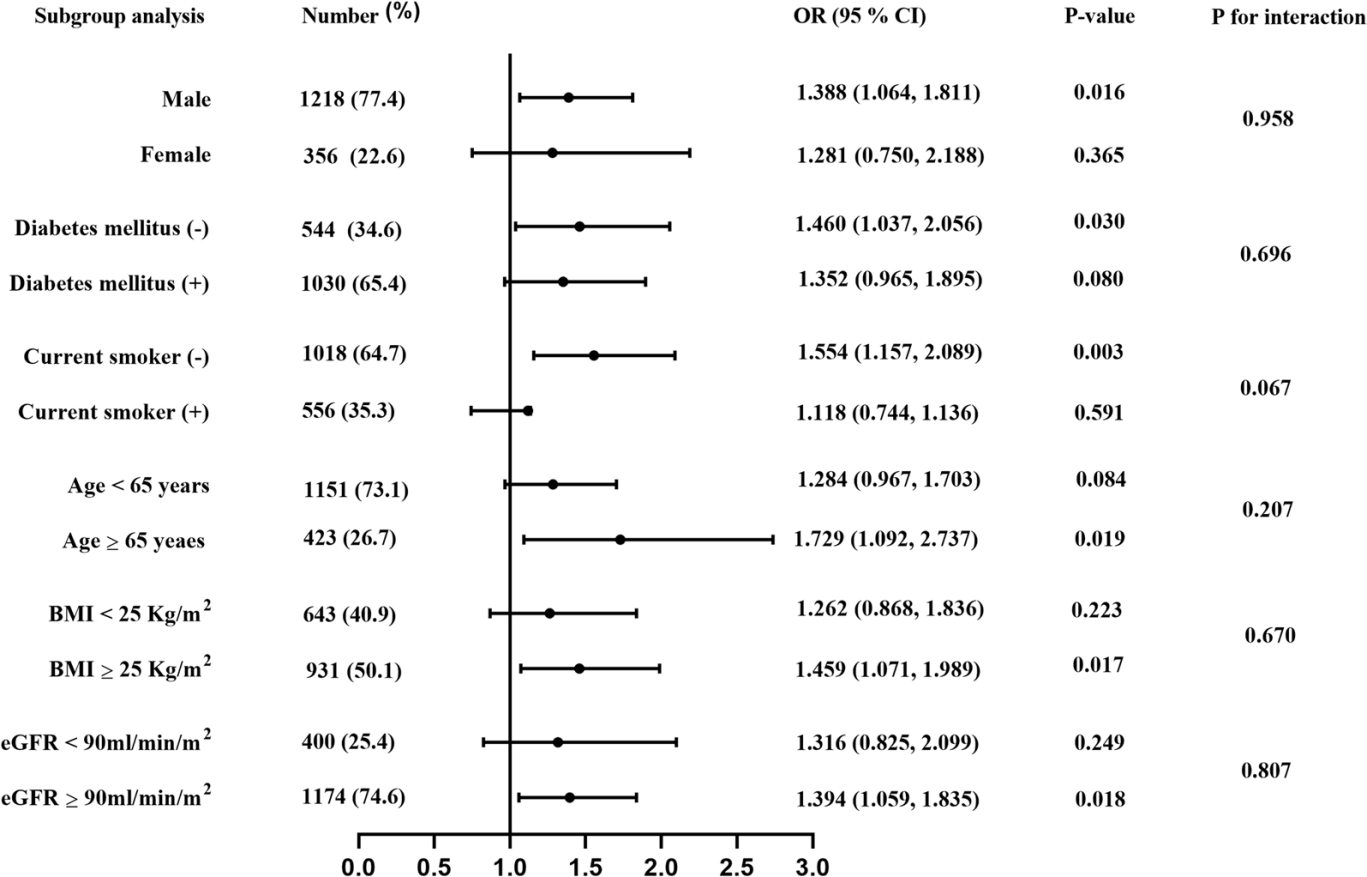
Forest plot investigating the association between the TyG index and the prevalence of DES-ISR in different subgroups. TyG index: triglyceride-glucose index; DES: drug-eluting stent; ISR: in-stent restenosis; BMI: body mass index; eGFR: estimated glomerular filtration rate; OR: odds ratio; CI: confidence interval

Subsequently, the independent association between the TyG index and DES-ISR was also assessed in various subgroups (Fig. 4). After adjustment for baseline variables with p < 0.1 in the univariate analysis, the positive association between the TyG index and the risk of DES-ISR was mainly reflected in the subgroups of age ≥ 65 years, male sex, eGFR ≥ 90 mL/min/1.73 m^2^, BMI ≥ 25 kg/m^2^, without DM, and noncurrent smokers. In addition, the trend was observed in subgroups of age < 65 years (p = 0.084) and those with DM (p = 0.08). Notably, a marginally significant (p = 0.067) interaction existed between the TyG index and smoking status with regard to the risk of DES-ISR.

### Incremental effects of the TyG index on the predictive value of DES-ISR

**Fig. 5:**
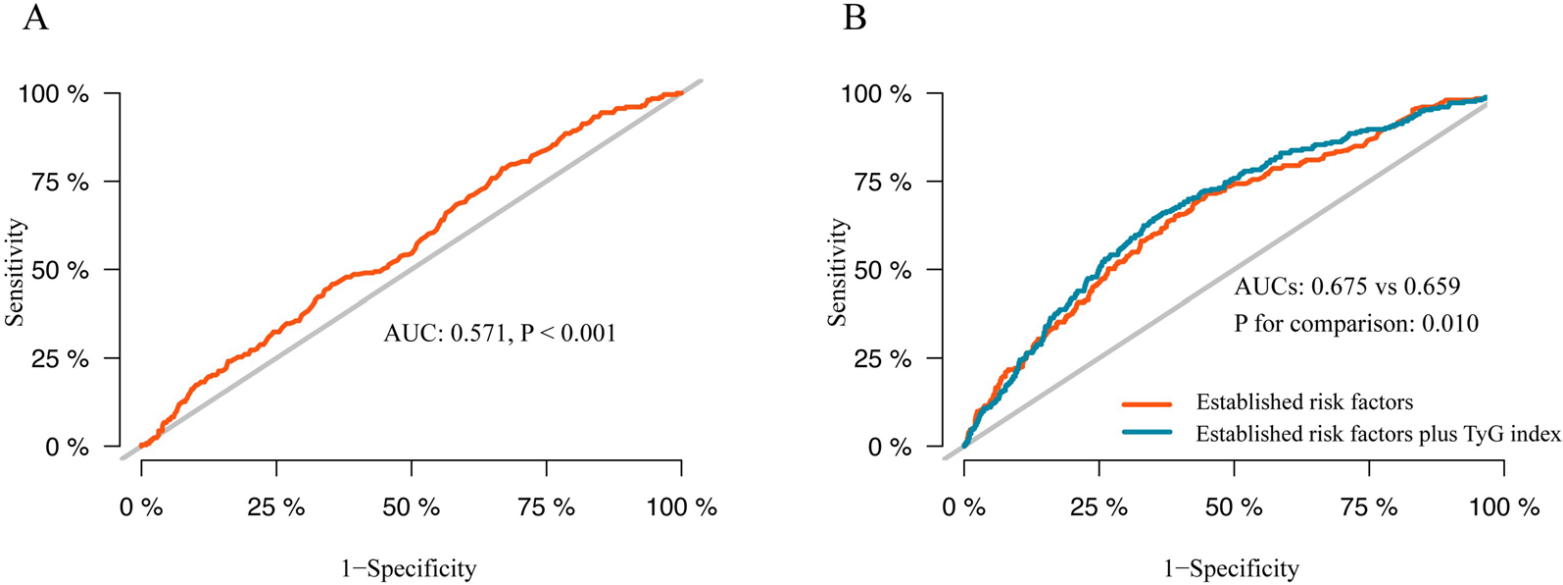
Receiver operating characteristic curve analysis of the TyG index to predict DES-ISR (**A**) and comparison of the C-statistics between the models (**B**). TyG index: triglyceride-glucose index; DES: drug-eluting stent; ISR: in-stent restenosis; AUC: area under curve

As presented in Fig. 5A, the ROC curve analysis revealed that the TyG index could provide mild predictive value for DES-ISR in patients with ACS who had an AUC of 0.571 (95% CI 0.533 to 0.608, p < 0.001). The optimal cut-off value was 8.55 (sensitivity: 78.7%, specificity: 33.2%). Meanwhile, as presented in Table 4, the C-statistic obtained from the model of established risk factors, which consisted of DM, previous PCI, target vessel in the LAD, target vessel in the RCA, and total length of stents, was 0.659 (95% CI 0.623 to 0.696, p < 0.001). Furthermore, Table 4 and Fig. 5B demonstrate that adding the TyG index to the model of established risk factors could lead to an increase in C-statistics (0.659 (0.623 to 0.696) vs 0.675 (0.639 to 0.711), p = 0.010), categorical NRI (0.090 (0.037 to 0.142), p < 0.001), and IDI (0.004 (0.0002 to 0.008), p = 0.040).

**Table 4:** Evaluate the predictive power of models for ISR after successful DES-based PCI

	C-statistic	P-value	P_for comparison_	Categorical NRI	P-value	IDI	P-value
Established risk factors	0.659 (0.623 to 0.696)	< 0.001	Ref		Ref		Ref
Established risk factors plus TyG index	0.675 (0.639 to 0.711)	< 0.001	0.010	0.090 (0.037 to 0.142)	< 0.001	0.004(0.0002 to 0.008)	0.040

ISR: in-stent restenosis; DES: drug-eluting stent; TyG index: triglyceride-glucose index; PCI: percutaneous coronary intervention; NRI: net reclassification improvement; IDI: integrated discrimination improvement

## Discussion

To the best of our knowledge, the present study was the first to investigate the association between IR assessed by the TyG index and DES-ISR in patients with ACS. The main findings of this study were as follows: (1) the TyG index, as a surrogate marker of IR, was significantly associated with cardiometabolic risk factors; (2) patients with a higher TyG index were more likely to have DES-ISR after successful PCI and vice versa; (3) the TyG index, either as a continuous or categorical variable, was independently associated with an increased risk of DES-ISR in the fully adjusted model; and (4) taking the TyG index into consideration may have clinical significance in optimizing the early risk stratification of DES-ISR in patients with ACS.

IR is a pathological condition characterized by defects in the uptake and utilization of glucose, which could lead to chronic hyperglycaemia and unique dyslipidaemia characterized by increased circulating TG and low concentrations of HDL-C [7]. Based on this theoretical background, the TyG index calculated from TG and FBG has been proposed as a surrogate indicator of IR [12, 13]. According to previous studies, the TyG index was significantly associated with IR assessed by the gold standard hyperinsulinaemic-euglycaemic clamp method and may even perform better than the HOMA among subjects with a wide range of body weights and glucose tolerances [14, 27]. In addition to better performance in the estimation of IR, the TyG index also correlated well with various metabolic abnormalities. Recent evidence has suggested that patients with a higher TyG index were more likely to have DM and hypertension, which is also demonstrated in our studies [15, 16]. Furthermore, in line with previous studies [21, 28], our present study also suggested that the TyG index was associated with other cardiometabolic risk factors.

In recent years, mounting clinical trials have also been designed to investigate the association of IR assessed by the TyG index with ASCVD. In the meta-analysis of cohort studies, Ding et al. reported that the TyG index, either as a continuous or categorical variable, was independently associated with the increased prevalence of ASCVD, and the association was not significantly affected by age, sex, or diabetes status in the subgroup analysis [29]. Meanwhile, a series of studies suggested that the TyG index may serve as a simple and effective tool in clinical practice to identify patients at risk of subclinical atherosclerosis, which is evaluated by the coronary artery calcium score, carotid ultrasound (intima-media thickness or plaque), and pulse wave velocity [28, 30–32]. Furthermore, recent evidence also indicated that the TyG index was not only positively associated with the rapid progression of coronary atherosclerosis and calcification but was also an independent risk factor for poor long-term prognosis in patients with stable CAD [19, 20, 33, 34]. Additionally, the positive association between the TyG index and adverse prognosis also persists in patients with ACS [21, 35], which is the leading cause of morbidity and mortality from cardiovascular disease worldwide [36]. Extending the above findings, our present work revealed that the TyG index was an independent predictor of DES-ISR in the entire study population. Based on our findings, novel therapeutic interventions to reduce the TyG index may be of importance for the prevention of DES-ISR in the future.

Subsequently, the stability of the association between the TyG index and DES-ISR was examined through subgroup analysis. This association was stable and persisted in most subgroups. Unexpectedly, this association seems to be more prominent in noncurrent smokers, and a non-significant interaction exist between the TyG index and current smoking status on DES-ISR. Although the mechanisms underlying this phenomenon remain unknown, it is necessary for us to take the current smoking status into consideration when we manage patients after PCI for ISR prevention, according to the TyG index.

Regarding the predictive power of the TyG index for CAD, accumulating evidence has demonstrated that the predictive value of the TyG index for CAD is mild to moderate, and introducing the TyG index into the Framingham model would improve the predictive value for CAD [37, 38]. Furthermore, Zhang et al. and Wang et al. reported that adding the TyG index into a baseline risk model could significantly improve the predictive accuracy for major adverse cardiac events (MACEs) in patients with ACS, although the predictive value of the TyG index for MACEs was mild [39, 40]. Consistent with previous studies, our present study suggested that introducing the TyG index into a model of established risk factors could improve our ability to identify patients at risk for DES-ISR. Although its incremental predictive value for DES-ISR was limited, considering a large and increasing number of patients ACS admitted for PCI every year [35, 36], it still seemed to be clinically important to perform assessments of the risk of DES-ISR combined with established risk factors.

The exact mechanisms underlying the close association of the TyG index with DES-ISR remain unknown. However, we speculated that TyG is a reliable marker of IR, which may mainly be due to the association. First, IR could aggravate the excessive proliferation of vascular smooth muscle cells via various potential signalling pathways, which is a prominent feature in the pathology of ISR [3, 41]. Second, IR could lead to endothelial dysfunction, which is a well-established risk factor for ISR [42, 43], by inducing inflammation, oxidative stress, and metabolic alterations [7, 44]. Third, the TyG index, as a surrogate marker of IR, was also strongly associated with the prevalence and progression of coronary artery calcification [28, 34], which could be another important mechanism. Finally, our present study and previous studies indicated that the TyG index, as a surrogate index of IR, was closely related to various cardiometabolic risk factors [21, 28, 34], which may account for the association.

Meanwhile, some limitations should be acknowledged in our present study. First, this is a single-centre, retrospective, and observational study. Therefore, this study could not determine the causality between the TyG index and DES-ISR. Second, our present study only included patients with ACS who underwent follow-up angiography within 6 to 24 months after PCI, which may cause selection bias and limit the generalizability of our findings to patients with chronic coronary syndrome. And the follow-up time was not long enough. Third, the identification of ISR in the present study mainly relied on visual assessment of angiography by 2 experienced cardiologists rather than more accurate and informative intracoronary imaging. Fourth, the present study fails to compare the role of HOMA-IR and the TyG index in DES-ISR, because fasting insulin is not routinely measured in our centre. Finally, the TyG index was evaluated only once after admission. Information on the change in the TyG index level during follow-up was limited.

## Conclusions

The TyG index, as a novel surrogate marker of IR, had an independent and positive association with the risk of ISR in patients with ACS after DES implantation. However, its incremental predictive value for DES-ISR was slight and needs to be further investigated. Additionally, multicentre, prospective, and randomized clinical trials are required in the future to investigate whether measures targeting the TyG index could provide favourable effects for the prevention of DES-ISR.

## Supplementary Information

**Additional file 1: Table S1.** Baseline characteristics of patients with and without ISR. **Table S2.** Association of DES-ISR and other clinical variables in the univariate analysis. **Figure S1.** Comparison of the TyG index between patients with or without ISR in the subgroups of sex (A), DM status (B), current smoking status (C), age (D), dichotomized baseline BMI (E), and dichotomized baseline eGFR (F). TyG index, triglyceride-glucose index; ISR, in-stent restenosis; DM, diabetes mellitus; BMI, body mass index; eGFR, estimated glomerular filtration rate.

## Data Availability

The datasets used/or analyzed during the current study are available from the corresponding author on reasonable request.

## Abbreviations

DESs: Drug-eluting stents
PCI: Percutaneous coronary intervention
ISR: In-stent restenosis
IR: Insulin resistance
ASCVD: Atherosclerotic cardiovascular disease
HOMA: Homeostatic model assessment
TyG index: Triglyceride-glucose index
TG: Triglyceride
FBG: Fasting blood glucose
DM: Diabetes mellitus
CAD: Coronary artery disease
ACS: Acute coronary syndrome
CABG: Coronary artery bypass grafting
BMS: Bare metal stent
eGFR: Estimated glomerular filtration rate
BMI: Body mass index
LVEF: Left ventricular ejection fraction
Hs-CRP: High sensitivity-C reactive protein
TC: Total cholesterol
LDL-C: Low-density lipoprotein-C
HDL-C: High-density lipoprotein-C
CTO: Chronic total occlusion
LAD: Left anterior descending artery
RCA: Right coronary artery
AUC: Area under curve
ROC: Receiver-operating characteristics
NRI: Net reclassification improvement
IDI: Integrated discrimination improvement
MACEs: Major adverse cardiac events
