# Supplementary material for: Triglyceride-glucose index is associated with in-stent restenosis in patients with acute coronary syndrome after percutaneous coronary intervention with drug-eluting stents
Source: Cardiovasc Diabetol. 2021 Jul 8;20:137. doi: 10.1186/s12933-021-01332-4 (PMC8268452; doi:10.1186/s12933-021-01332-4)
Supplement: Supplementary file 1 — Additional file 1: Table S1. Baseline characteristics of patients with and without ISR. Table S2. Association of DES-ISR and other clinical variables in the univariate analysis. Figure S1. Comparison of the TyG index between patients with or without ISR in the subgroups of sex (A), DM status (B), current smoking status (C), age (D), dichotomized baseline BMI (E), and dichotomized baseline eGFR (F). TyG index, triglyceride-glucose index; ISR, in-stent restenosis; DM, diabetes mellitus; BMI, body mass index; eGFR, estimated glomerular filtration rate. [file 12933_2021_1332_MOESM1_ESM.docx]

Table S1. Baseline characteristics of patients with and without ISR

|  | ISR group  (n = 253) | Non-ISR group  (n = 1321) | P-value |
| --- | --- | --- | --- |
| Age, years | 58.95 ± 9.81 | 58.29 ± 9.32 | 0.310 |
| Male, n (%) | 195 (77.1) | 1023 (77.4) | 0.898 |
| BMI, kg/m^2^ | 26.05 ± 2.90 | 25.94 ± 3.29 | 0.617 |
| LVEF, % | 61.23 ± 7.92 | 62.34 ± 6.81 | 0.050 |
| **Diagnosis, n (%)** |  |  | 0.995 |
| UA | 214 (84.6) | 1120 (84.8) |  |
| NSTEMI | 19 (7.5) | 97 (7.3) |  |
| STEMI | 20 (7.9) | 104 (7.9) |  |
| **Medical history, n (%)** |  |  |  |
| Current smoking | 90 (35.6) | 466 (35.3) | 0.928 |
| Hypertension | 176 (69.6) | 840 (63.6) | 0.069 |
| Hypercholesteraemia | 95 (37.5) | 515 (39.0) | 0.668 |
| Diabetes mellitus | 118 (46.6) | 426 (32.2) | <0.001 |
| Previous stroke | 23 (9.1) | 104 (7.9) | 0.515 |
| Previous PCI | 69 (27.3) | 220 (16.7) | <0.001 |
| **Laboratory tests** |  |  |  |
| Hs-CRP, mg/L | 1.93 (0.69, 4.90) | 1.33 (0.53, 3.78) | 0.003 |
| eGFR, ml/min/1.73m^2^ | 96.62 ± 14.79 | 96.52 ± 14.72 | 0.920 |
| Uric acid, umol/L | 339.78 ± 77.14 | 346.58 ± 86.04 | 0.242 |
| Homocysteine, umol/L | 12.20 (9.55, 15.70) | 12.50 (9.70, 16.10) | 0.452 |
| FBG, mmol/L | 7.27 ± 2.86 | 6.43 ± 2.16 | <0.001 |
| Triglycerides, mmol/L | 1.48 (1.10, 2.00) | 1.39 (1.01, 1.98) | 0.152 |
| TC, mmol/L | 4.19 ± 1.07 | 4.10 ± 1.07 | 0.215 |
| HDL-C, mmol/L | 1.06 ± 0.24 | 1.07 ± 0.24 | 0.344 |
| LDL-C, mmol/L | 2.54 ± 0.94 | 2.46 ± 0.88 | 0.166 |
| **Angiography** |  |  |  |
| LM disease, n (%) | 27 (10.7) | 112 (8.5) | 0.260 |
| Multivessel/LM disease, n (%) | 193 (76.3) | 1002 (75.9) | 0.883 |
| Chronic total occlusion, n (%) | 79 (31.2) | 288 (21.8) | 0.001 |
| SYNTAX score | 14.92 ± 7.30 | 13.74 ± 7.43 | 0.021 |
| **Intervention** |  |  |  |
| Target vessel, n (%) |  |  |  |
| LM | 16 (6.3) | 62 (4.7) | 0.274 |
| LAD | 160 (63.2) | 729 (55.2) | 0.018 |
| LCX | 73 (28.9) | 362 (27.4) | 0.637 |
| RCA | 118 (46.6) | 522 (39.5) | 0.035 |
| Intracoronary imagine, n (%) | 9 (3.6) | 91 (6.9) | 0.047 |
| DES-sirolimus, n (%) | 152 (60.1) | 697 (52.8) | 0.032 |
| DES-zotarolimus, n (%) | 61 (24.1) | 284 (21.5) | 0.358 |
| DES-everolimus, n (%) | 93 (36.8) | 504 (38.2) | 0.675 |
| Number of stents, / patients | 2 (1, 3) | 1 (1, 2) | <0.001 |
| Multiple stents (≥2) | 149 (58.9) | 640 (48.4) | 0.002 |
| Total length of stents, mm/patients | 45.00 (28.00, 76.50) | 36.00 (22.00, 60.00) | <0.001 |
| Minimal stent diameter, mm | 2.75 ± 0.42 | 2.86 ± 0.47 | <0.001 |
| **Medication used at Discharge, n (%)** |  |  |  |
| Aspirin | 253 (100.0) | 1321 (100.0) | >0.999 |
| Clopidogrel/Ticagrelor | 253 (100.0) | 1321 (100.0) | >0.999 |
| Statin | 252 (99.6) | 1318 (99.8) | 0.626 |
| β-block | 178 (70.4) | 910 (68.9) | 0.655 |
| ACEI/ARB | 128 (50.6) | 594 (45.0) | 0.100 |
| Insulin | 43 (17.0) | 131 (9.9) | 0.001 |
| Oral hypoglycemic agents | 98 (38.7) | 333 (25.2) | <0.001 |

ISR, in-stent restenosis; BMI, body mass index; LVEF, left ventricular ejection fraction; UA, unstable angina; NSTEMI, non ST-segment elevation myocardial infarction; STEMI, ST-segment elevation myocardial infarction; PCI, percutaneous coronary intervention; Hs-CRP, high sensitivity-C reactive protein; eGFR, estimated glomerular filtration rate; FBG, fasting blood glucose; TC, total cholesterol; HDL-C, high-density lipoprotein-C; LDL-C, low-density lipoprotein-C; LM, left main artery; LAD, left anterior descending artery; LCX, left circumflex artery; RCA, right coronary artery; DES, drug-eluting stent; ACEI/ARB, angiotensin-converting enzyme inhibitor/angiotensin receptor blocker.

Table S2. Association of DES-ISR and other clinical variables in the univariate analysis.

| Variables | OR | 95 % CI | P value |
| --- | --- | --- | --- |
| LVEF | 0.980 | 0.962 to 0.998 | 0.030 |
| Hypertension | 1.309 | 0.979 to 1.750 | 0.069 |
| Diabetes mellitus | 1.836 | 1.398 to 2.412 | <0.001 |
| Previous PCI | 1.877 | 1.373 to 2.565 | <0.001 |
| SYNTAX | 1.021 | 1.003 to 1.039 | 0.022 |
| Target in LAD | 1.397 | 1.058 to 1.844 | 0.018 |
| Target in RCA | 1.338 | 1.021 to 1.754 | 0.035 |
| Intracoronary imagine | 0.499 | 0.248 to 1.002 | 0.051 |
| DES-sirolimus | 1.347 | 1.025 to 1.772 | 0.033 |
| Total length of stents, mm/patients | 1.010 | 1.006 to 1.014 | <0.001 |
| Minimal stent diameter, mm | 0.570 | 0.415 to 0.784 | 0.001 |

DES, drug-eluting stent; ISR, in-stent restenosis; LVEF, left ventricular ejection fraction; PCI, percutaneous coronary intervention; LAD, left anterior descending artery; RCA, right coronary artery.

Figure S1


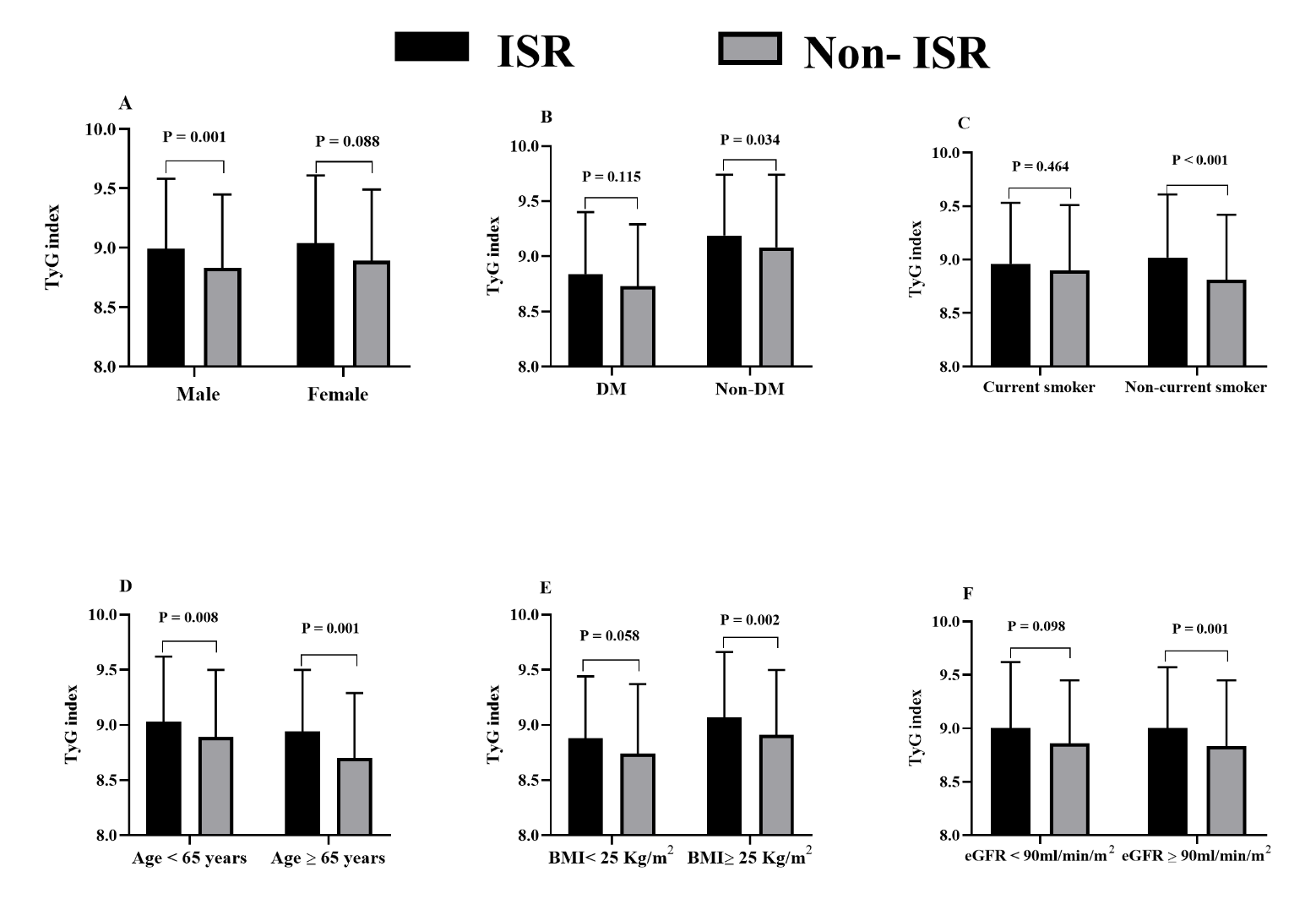


Comparison of the TyG index between patients with or without ISR in the subgroups of sex (A), DM status (B), current smoking status (C), age (D), dichotomized baseline BMI (E), and dichotomized baseline eGFR (F). TyG index, triglyceride-glucose index; ISR, in-stent restenosis; DM, diabetes mellitus; BMI, body mass index; eGFR, estimated glomerular filtration rate.
